# Reduced sTWEAK and Increased sCD163 Levels in HIV-Infected Patients: Modulation by Antiretroviral Treatment, HIV Replication and HCV Co-Infection

**DOI:** 10.1371/journal.pone.0090541

**Published:** 2014-03-04

**Authors:** Luis M. Beltrán, Rocío Muñoz Hernández, Rebeca S. de Pablo Bernal, José S. García Morillo, Jesús Egido, Manuel Leal Noval, Sara Ferrando-Martinez, Luis M. Blanco-Colio, Miguel Genebat, José R. Villar, Rafael Moreno-Luna, Juan Antonio Moreno

**Affiliations:** 1 Instituto de Biomedicina de Sevilla, Hospital Universitario Virgen del Rocío/Centro Superior de Investigaciones Científicas/Universidad de Sevilla, Unidad Clínico-Experimental de Riesgo Vascular, Sevilla, Spain; 2 Unidad Metabólico-Vascular, Fundación de Investigación IdiPAZ-Hospital Universitario La Paz, Madrid, Spain; 3 Laboratorio de Inmunovirología, Unidad Clínica de Enfermedades Infecciosas, Microbiología y Medicina Preventiva, Instituto de Biomedicina de Sevilla, Hospital Universitario Virgen del Rocío/Centro Superior de Investigaciones científicas/Universidad de Sevilla, Sevilla, Spain; 4 Laboratorio de Patología Vascular, Instituto de Investigación Sanitaria-Fundación Jiménez Díaz, Madrid, Spain; 5 Spanish Biomedical Research Centre in Diabetes and Associated Metabolic Disorders, Madrid, Spain; 6 Laboratorio Inmunobiología Molecular, Hospital General Universitario Gregorio Marañón, Madrid, Spain; 7 Instituto de Investigación Sanitaria Gregorio Marañón, Madrid, Spain; 8 Networking Research Center on Bioengineering, Biomaterials and Nanomedicine, Madrid, Spain; 9 Department of Vascular Physiopathology, Hospital Nacional de Parapléjicos, Servicio de Salud de Castilla La Mancha, Toledo, Spain; University of Cape Town, South Africa

## Abstract

**Background:**

Patients infected with the human immunodeficiency virus (HIV) have an increased risk of cardiovascular disease due to increased inflammation and persistent immune activation. CD163 is a macrophage scavenger receptor that is involved in monocyte-macrophage activation in HIV-infected patients. CD163 interacts with TWEAK, a member of the TNF superfamily. Circulating levels of sTWEAK and sCD163 have been previously associated with cardiovascular disease, but no previous studies have fully analyzed their association with HIV.

**Objective:**

The aim of this study was to analyze circulating levels of sTWEAK and sCD163 as well as other known markers of inflammation (hsCRP, IL-6 and sTNFRII) and endothelial dysfunction (sVCAM-1 and ADMA) in 26 patients with HIV before and after 48 weeks of antiretroviral treatment (ART) and 23 healthy subjects.

**Results:**

Patients with HIV had reduced sTWEAK levels and increased sCD163, sVCAM-1, ADMA, hsCRP, IL-6 and sTNFRII plasma concentrations, as well as increased sCD163/sTWEAK ratio, compared with healthy subjects. Antiretroviral treatment significantly reduced the concentrations of sCD163, sVCAM-1, hsCRP and sTNFRII, although they remained elevated when compared with healthy subjects. Antiretroviral treatment had no effect on the concentrations of ADMA and sTWEAK, biomarkers associated with endothelial function. The use of protease inhibitors as part of antiretroviral therapy and the presence of HCV-HIV co-infection and/or active HIV replication attenuated the ART-mediated decrease in sCD163 plasma concentrations.

**Conclusion:**

HIV-infected patients showed a proatherogenic profile characterized by increased inflammatory, immune-activation and endothelial-dysfunction biomarkers that partially improved after ART. HCV-HIV co-infection and/or active HIV replication enhanced immune activation despite ART.

## Introduction

Patients with human immunodeficiency virus (HIV) infection have a higher risk of developing cardiovascular disease than does the general population [1]–[3]. Several potential mechanisms may explain the relationship between HIV infection and atherosclerosis. HIV infection induces persistent inflammation and immune activation, endothelial dysfunction, lipid disorders and direct vascular injury [4]. Furthermore, although antiretroviral therapy (ART) decreases morbidity and mortality in HIV patients [5], a number of studies have also reported long-term adverse effects of ART on vasculature, including oxidative stress and endothelial dysfunction [6]. Conventional risk prediction models based on traditional cardiovascular risk factors may underestimate the incidence of atherosclerotic cardiovascular events in patients with HIV infection [7] because they do not consider the specific atherosclerotic processes reported by these patients. Identifying biomarkers for HIV patients at higher cardiovascular risk may be of great interest and could improve cardiovascular risk predictions by traditional stratification scales.

Tumor necrosis factor (TNF)-like weak inducer of apoptosis (TWEAK) is a member of the TNF superfamily that is synthesized as a type II transmembrane glycoprotein and circulates in plasma as a soluble form (sTWEAK) [8]. TWEAK may participate in the development of atherosclerosis by promoting the production of proinflammatory cytokines and altering the proliferation and migration of vascular smooth muscle cells and the expression of extracellular matrix-degrading enzymes [9]–[11]. sTWEAK plasma levels are decreased in patients with peripheral artery disease (PAD) [12], coronary artery disease (CAD) [13], carotid atherosclerosis [14], abdominal aortic aneurysms [15] and atherosclerosis-associated disorders such as type 2 diabetes or chronic kidney disease [16]–[18]. Moreover, sTWEAK levels are associated with an adverse prognosis in patients with chronic stable heart failure, myocardial infarction, PAD or non-dialysis CKD [19]–[22].

CD163 has been recently described as a new receptor for TWEAK present on the surface of monocytes/macrophages [23]–[24]. A soluble form (sCD163) is generated by the proteolytic cleavage of CD163 at the cell surface in response to proinflammatory and oxidative stimuli [25]–[27]. The plasma levels of sCD163 are increased in patients with CAD [28], carotid atherosclerosis [24], PAD [12] and atherosclerosis-associated disorders such as type 2 diabetes [29]. The plasma levels of sCD163 have been associated with HIV disease activity in early and chronically infected patients [30]. Moreover, sCD163 plasma levels have been independently associated with noncalcified coronary atherosclerotic plaques in HIV-infected men [31] and correlate with arterial inflammation as determined by positron emission tomography [32]. To our knowledge, there are no studies examining sTWEAK plasma levels in patients with HIV infection. In this study, we analyzed sTWEAK and sCD163 plasma levels and other known markers of inflammation (high-sensitivity C-reactive protein [hsCRP], interleukin 6 [IL-6] and soluble tumor necrosis alpha receptor II [sTNFRII]), endothelial dysfunction (soluble vascular cell adhesion molecule 1 [sVCAM-1] and asymmetric dimethylarginine [ADMA]) and thrombotic activity (D-dimer) in healthy subjects and patients with HIV. Furthermore, we assessed the influence of antiretroviral treatment on these biomarkers after 48 weeks of follow-up.

## Materials and Methods

### Subjects and design

Twenty-three individuals without HIV infection (control subjects) and 26 patients infected with HIV were included in the study. HIV patients received ART according to regional recommendations for initiating ART [33]. Patients included in the HIV group were 18 years or older, had a documented HIV-1 infection at least 6 months before entering the study and had not received any antiretroviral drug before the baseline date. Regardless of their HIV status, subjects were excluded if they had known cardiovascular disease, diabetes mellitus, chronic kidney disease, pregnancy, active malignancy or any terminal medical condition. Blood samples were obtained from HIV patients at baseline and 48 weeks after initiating ART. Blood samples were obtained only once from the participants in the control group. This study was conducted at Virgen del Rocío University Hospital and the Biomedicine Institute of Seville (IBiS) (Seville, Spain).

### Ethics statement

The study was conducted according to the principles expressed in the Declaration of Helsinki. Patients and controls provided written informed consent, and the Ethical Committee of the Virgen del Rocío University Hospital approved the study.

### Laboratory investigation

Venous blood was collected in EDTA tubes. The whole-plasma samples were stored at −80°C until analyses were performed. Laboratory analyses (lipid, glucose, creatinine, CD4 and CD8 cells, HIV copies and RNA levels) were performed according to routine practice in our hospital. sTWEAK and sCD163 plasma concentrations were measured in duplicate with commercially available ELISA kits (Bender MedSystems, [Vienna, Austria]; and R&D Systems [Abingdon, UK], respectively). hsCRP, sTNFRII, sVCAM-1 and ADMA levels were also determined with commercially available ELISA kits (Immundiagnostik AG [Bensheim, Germany], R&D Systems [Abingdon, UK], DLD Diagnostika GmbH [Hamburg, Germany] and eBioscience's [San Diego, USA], respectively). IL-6 was measured by ultrasensitive ELISA (Quantikine HS Human IL-6 Immunoassay; R&D Systems [Abingdon, UK]). D dimer levels were measured in an automated latex-enhanced immunoassay (HemosIL D-Dimer HS 500; Instrumentation Laboratory [Bedford, UK]). Ig G antibodies to cytomegalovirus were determined by enzyme immunoassay (GenWay Biotech [San Diego, USA]).

### Statistical analysis

The data are presented as the mean ± standard deviation or the median (interquartile range) for continuous variables depending on the normality of the distribution. Categorical variables are presented as proportion (count). To test whether the variables were normally distributed, we performed the Shapiro-Wilk test. Qualitative variables were analyzed using Fisher's test and the χ^2^ test. The comparisons between two groups were performed using Student's t-test for normally distributed continuous variables. The Mann-Whitney U test and Wilcoxon matched-pairs test were used to determine significant difference for unpaired and paired data, respectively, if the distribution was non-normal. Pearson correlation coefficients were used to assess correlations for normally distributed data. For non-normally distributed end points, data were either log-transformed, or Spearman rho was used to assess correlation. We conducted a multivariate linear regression analysis in order to evaluate the relative impact of different baseline characteristics (age, triglycerides, BMI, antihypertensive treatment and cytomegalovirus IgG positivity) on plasma sTWEAK, sCD163 and sCD163/sTWEAK levels. *P*<0.05 was considered statistically significant. Statistics were calculated using the Statistics Package for Social Sciences software (SPSS for Mac OS, version 20.0).

## Results

### HIV case-control study

The general characteristics of the study subjects are presented in **Table 1**. The median time from HIV diagnosis was 5 months (IQR 1–26). The route of HIV infection was heterosexual in 6 patients, homosexual in 15 patients and intravenous drug use in 5 cases. Three patients presented with hepatitis C virus (HCV)-HIV co-infection, and they did not receive specific treatment for HCV during the study. Five patients received anti-hypertensive treatment, and one patient was treated for dyslipidemia. No HCV infection, hypertension or dyslipidemia was reported in the control group. Of the 26 patients, 12 received protease inhibitors (PIs), 23 received nucleoside reverse transcriptase inhibitors (NRTIs), 18 received non-nucleoside reverse transcriptase inhibitors (NNRTIs) and 3 received maraviroc during their treatment.

**Table 1:** Cardiovascular-related, immunovirologic and biomarkers data in control subjects and HIV-infected patients at baseline and 48 weeks after initiating ART.

	Control (n = 23)	Naïve HIV(n = 26)	*p^a^*	ART HIV (n = 26)	*p^b^*	*p^c^*
Age (years)	32.1±8	37.5±10.3	**0.05**	-	-	-
Sex (males/females)	12/11	20/6	0.065	-	-	-
Smokers (n)	7	13	NS	-	-	-
Treatment for hypertension (%)	0	19.2	**0.026**	-	-	-
BMI (kg/m^2^)	23.7±2.3	25.8±4.5	0.07	-	-	-
Total cholesterol (mg/dL)	185.7±29.5	170.8±45.3	NS	197.1±43.1	NS	**0.001**
LDL cholesterol (mg/dl)	115.6±31.7	103.3±37.2	NS	109.6±34.8	NS	NS
HDL c	62 (60–71)	42 (36–52)	**<0.001**	50 (41–68)	**0.01**	**0.006**
LDLc/HDLc ratio	1.72±0.58	2.45±0.95	0.02	2.19±1.01	NS	NS
Triglycerides (mg/dL)	60 (50–91)	110 (75–142)	**0.001**	123 (89–197)	**<0.001**	**0.039**
Creatinine (mg/dL)	0.82±0.11	0.79±0.15	NS	0.83±0.13	NS	NS
Fasting glucose (mg/dL)	83±6.4	89.3±10.7	0.025	93.1±10	**0.002**	NS
CD4 (cells/µL)	-	283 (151–405)	-	435 (300–652)	-	**<0.001**
CD8 (cells/µL)	-	743 (570–1130)	-	758 (563–1077)	-	NS
HIV-RNA <20 (n/total)^d^	-	NA	-	23/26	-	NA
HIV-RNA (copies/mL)	-	32050 (8520–171246)	-	248 (173–759)^ e^	-	NA^e^
CMV IgG+ (%)	47.6	95	**0.001**	-	-	-
sVCAM-1 (ng/mL)	415 (307–601)	617 (424–832)	**0.011**	393 (318–519)	NS	**<0.001**
ADMA (ng/mL)	79.5 (43–112)	210 (158–290)	**<0.001**	194 (166–256)	**<0.001**	NS
hsCRP (mg/L)	0.4 (0.21–1.33)	2.41 (1.62–5.43)	**<0.001**	1.16 (0.59–1.58)	**0.025**	**0.002**
IL-6 (pg/mL)	0.42 (0.3–0.74)	1.02 (0.6–1.43)	**0.001**	0.58 (0.36–1.2)	NS	**0.02**
sTNFRII (pg/mL)	1923(1586–2300)	4553 (3051–6671)	**0.001**	2363 (1782–3930)	**0.003**	**0.001**
D-dimer (µg/L)	287 (220–412)	306 (212–582)	NS	269 (212–457)	NS	NS

BMI =  Body mass index. CMV = cytomegalovirus, NS =  Not significant. NA =  Not applicable. a) *p* for control subjects vs. naive HIV patients. b) *p* for control subjects vs. HIV patients with 48 weeks of ART. c) *p* for comparison of HIV patients at baseline vs. 48 weeks. d) Proportion of HIV patients with undetectable viral load at 48 weeks. e) Values calculated using the only 3 patients with detectable viral loads at 48 weeks (with descriptive aims, no comparison).

**Figure 1:**
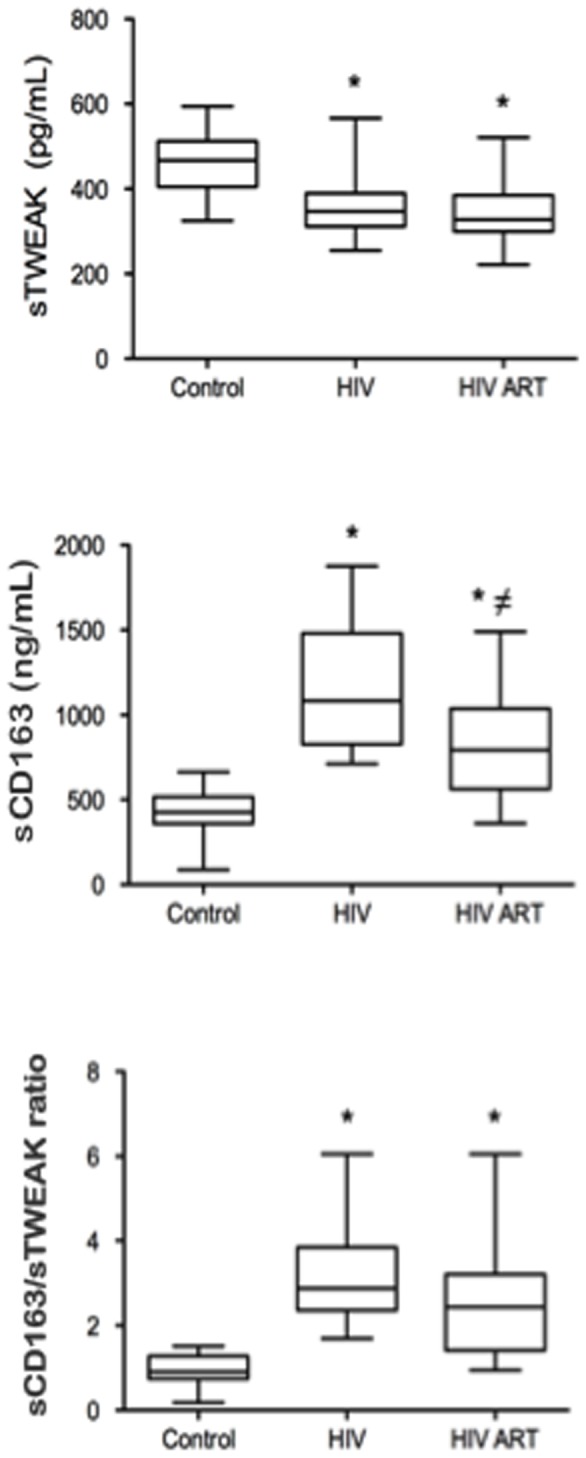
Comparison of sTWEAK, sCD163 and the sCD163/sTWEAK ratio. sTWEAK, sCD163 and the sCD163/sTWEAK ratio in control subjects and HIV patients at baseline and after ART. *P<0.05 vs. control, ≠ *P*<0.05 vs. HIV baseline.

HIV-infected patients were older than the control individuals (37.5±10.3 vs. 32.1±8 years; *P* = 0.05) and had a higher proportion of positive IgG antibodies to cytomegalovirus (95% vs. 47.6%; *P* = 0.001). The proportion of males, active smokers and body mass index (BMI) did not significantly differ between groups. At baseline, HIV-infected patients had significantly higher levels of glucose, triglycerides and the LDL/HDL ratio in addition to lower levels of HDL than controls (**Table 1**). HIV patients at baseline also had elevated concentrations of sVCAM-1, ADMA, hsCRP, IL-6 and sTNFRII (**Table 1**). No differences were found for D-dimer. sTWEAK levels were significantly lower in HIV-infected vs. control patients [354 (329–447) vs. 468 (410–512) pg/ml; *P* = 0.001], whereas sCD163 and sCD163/sTWEAK ratio were higher in HIV-infected patients compared with control subjects [1,085 (828–1,480) vs. 448 (362–526) ng/ml; *P*<0.001 and 2.88 (2.37–3.85) vs. 0.94 (0.78–1.29); *P*<0.001, respectively] (**Figure 1**). Differences in sTWEAK, sCD163 and sCD163/sTWEAK levels remained significant after adjusting for age, triglycerides, BMI, antihypertensive treatment and cytomegalovirus IgG positivity (*P* = 0.012; *P*<0.001 and *P*<0.001 respectively). Age and anti-hypertensive therapy were independently associated with the sCD163 plasma concentration and the sCD163/sTWEAK ratio, but not with sTWEAK levels.

### Effect of antiretroviral treatment on HIV patients

ART reduced hsCRP, IL-6, sTNFRII and sVCAM-1 plasma levels, but no differences were observed in ADMA or D-dimer concentrations (**Table 1**). ART did not modify the sTWEAK levels [351 (318–401) vs. 325 (299–386) pg/ml; *P* = 0.292). However, the sCD163 levels of HIV-infected patients decreased significantly after 48 weeks of ART [1,085 (828–1,480) vs. 792 (562–1,025) ng/ml; *P* = 0.02], and the sCD163/sTWEAK ratio showed a decreasing trend [2.88 (2.37–3.85) vs. 2.44 (1.42–3.2); *P* = 0.062] after ART (**Figure 1**).

We then determined whether ART reestablished inflammatory biomarkers in HIV-infected patients compared with controls. Despite the use of ART, HIV-infected patients had higher concentrations of ADMA, hsCRP and sTNFRII than did the controls. There were no differences in sVCAM-1, IL-6 or D-dimer levels (**Table 1**). Similarly, sCD163 and sCD163/sTWEAK levels remained elevated despite treatment in HIV-infected patients after ART compared with controls [792 (562–1,025) vs. 448 (362–526) ng/ml; *P*<0.001 and 2.44 (1.42–3.2) vs. 0.94 (0.78–1.29); *P*<0.001, respectively). sTWEAK levels were lower in HIV-infected patients after ART compared with control subjects [325 (299–386) vs. 468 (410–512) pg/ml; *P*<0.001, respectively].

We then analyzed the influence of the type of treatment on the evolution of the different biomarkers analyzed. Patients given protease inhibitors (PIs) in their treatment (n = 10) had a smaller decrease in sCD163 plasma levels compared with patients that did not receive PIs [203.7 (−253 to 441.5) vs. 507.8 (107.4 to 728.4) ng/ml; *P* = 0.047, respectively]. No significant differences were observed in other biomarkers or when we analyzed the influence of other antiretroviral drugs (nucleoside reverse transcriptase inhibitors, non-nucleoside reverse transcriptase inhibitors and maraviroc) (data not shown).

### Influence of HCV-HIV co-infection and active viral replication

After 48 weeks of ART, 23 of the 26 HIV-infected patients had undetectable viral loads with a median CD4 cell count of 435 cells/ml (IQR 300–652). The median viral load in the 3 patients with active HIV replication was 248 copies/ml (IQR 173–759). Because HCV-HIV co-infection and active HIV replication may maintain immune activation, we analyzed the possible influence of these conditions on all the biomarkers analyzed. Patients with HCV-HIV co-infection and/or active HIV replication after 48 weeks on ART (n = 5) had a significantly increased sCD163 concentration and sCD163/sTWEAK ratio compared with patients without HCV-HIV co-infection and controlled viral replication (n = 21) [1,290 (IQR 997–2,152) vs. 776 (IQR 501–951) ng/ml; *P* = 0.01 and 4.08 (IQR 3.04–9.01) vs. 2.15 (IQR 1.3–2.71); *P*<0.001, respectively]. No differences were found in the sTWEAK or ADMA levels according to the presence of HCV and active HIV replication. However, a trend toward a worse inflammatory profile with higher hsCRP [2.14 (IQR 0.89–9.6) vs. 0.99 (IQR 0.48–1.53) mg/l, *P* = 0.11] and sTNFRII [4,404 (IQR 2,060–4,825) vs. 2,203 (IQR 1,741–2,977) pg/ml, *P* = 0.13] was observed between patients with HCV-HIV co-infection and/or active HIV replication compared to those without these conditions.

Differences observed between HIV patients at baseline versus after ART for hsCRP [2.18 (IQR 1.23–3.65) vs. 0.99 (IQR 0.48–1.53) mg/l, *P*<0.001, respectively], IL-6 [1.03 (IQR 0.6–2.32) vs. 0.43 (IQR 0.32–0.89) pg/ml, *P* = 0.041, respectively], sTNFRII [4,453 (IQR 3,003–6,791) vs. 2,170 (IQR 1,737–2,840) pg/ml, *P* = 0.008, respectively], sCD163 plasma levels [1,079 (IQR 865–1,416) vs. 776 (IQR 501–951) ng/ml; *P* = 0.002, respectively] and the sCD163/sTWEAK ratio [2.87 (IQR 2.47–3.36) vs. 2.15 (IQR 1.3–2.71); *P* = 0.005, respectively] were also significant when patients with HCV-HIV co-infection or active HIV replication were excluded from the analysis.

### Correlations between sTWEAK and sCD163 with HIV infection markers

Correlations between sTWEAK, sCD163 and sCD163/sTWEAK with immunovirologic and metabolic parameters are presented in **Table 2**. sCD163 and the sCD163/sTWEAK ratio was inversely correlated with CD4 nadir at baseline (r = −0.473; *P* = 0.015 and r = −0.437; *P* = 0.025, respectively) and with CD4 total count after ART (r = −0.562; *P* = 0.003 and r = −0.527; *P* = 0.006, respectively). We did not find a significant correlation between these markers and viral load in HIV-infected patients at baseline or after ART. sTWEAK correlated inversely with hsCRP (r = −0.471; *P*<0.001), whereas sCD163 and the sCD613/sTWEAK ratio correlated positively with ADMA (r = 0.689; *P*<0.001 and r = 0.615; *P*<0.001, respectively), sVCAM-1 (r = 0.374; *P* = 0.002 and r = 0.441; *P*<0.001, respectively) hsCRP (r = 0.445; *P*<0.001 and r = 0.557; *P*<0.001, respectively), IL-6 (r = 0.468; *P* = 0.038 and r = 0.471; *P* = 0.036) and sTNFRII (r = 0.568; *P* = 0.009 and r = 0.567; *P* = 0.009) at baseline.After antiretroviral treatment sCD163 and sCD163/sTWEAK ratio correlated positively with IL-6 (r = 0.529; *P* = 0.008 and r = 0.412; *P* = 0.045) and sTNFRII (r = 0.576; *P* = 0.003 and r = 0.559; *P* = 0.005), but not with the other biomarkers.

**Table 2:** Correlation between sTWEAK, sCD163 and sCD163/sTWEAK with immunovirological, inflammatory and endothelial biomarkers.

	*sTWEAK*		*sCD163*		*sCD163/sTWEAK*	
	Baseline	ART	Baseline	ART	Baseline	ART
Age	0.062; *p* = 0.762	−0.145; *p* = 0.479	0.348; *p* = 0.081	0.200; *p* = 0.327	0.277; *p* = 0.171	0.254; *p* = 0.211
Viral load	−0.138; *p* = 0.502	−0.12; *p* = 0.955	0.216; *p* = 0.288	0.295; *p* = 0.143	0.207; *p* = 0.311	0.258; *p* = 0.203
CD4 nadir	−0.112; *p* = 0.587	−0.346; *p* = 0.083	**−0.473; ** ***p*** ** = 0.015**	−0.346; *p* = 0.083	**−0.437; ** ***p*** ** = .025**	−0.232; *p* = 0.254
CD4 count	−0.136; *p* = 0.509	0.064; *p* = 0.756	−0.361; *p* = 0.07	**−0.562; ** ***p*** ** = 0.003**	−0.288; *p* = 0.154	**−0.527; ** ***p*** ** = 0.006**
CD8 count	−0.061; *p* = 0.766	−0.044; *p* = 0.831	−0.132; *p* = 0.522	−0.181; *p* = 0.377	−0.111; *p* = 0.589	−0.144; *p* = 0.483
CD4/CD8	−0.227; *p* = 0.265	−0.030; *p* = 0.883	−0.283; *p* = 0.162	**−0.393; ** ***p*** ** = 0.047**	−0.169; *p* = 0.410	−0.346; *p* = 0.083
TChol	0.175; *p* = 0.392	0.095; *p* = 0.644	−0.108; *p* = 0.600	−0.207; *p* = 0.311	−0.299; *p* = 0.137	−0.268; *p* = 0.185
LDL	0.252; *p* = 0.224	0.112; *p* = 0.588	−0.121; *p* = 0.564	−0.364; *p* = 0.067	−0.334; *p* = 0.103	**−0.396; ** ***p*** ** = 0.045**
HDL	**−0.454; ** ***p*** ** = 0.023**	**−0.400; ** ***p*** ** = 0.043**	−0.082; *p* = 0.698	0.313; *p* = 0.119	0.159; *p* = 0.447	**0.446; ** ***p*** ** = 0.022**
Triglycerides	0.179; *p* = 0.382	**0.530; ** ***p*** ** = 0.005**	−0.001; *p* = 0.996	−0.138; *p* = 0.500	−0.183; *p* = 0.371	−0.352; *p* = 0.077
Creatinine	0.035; *p* = 0.867	0.012; *p* = 0.952	**−0.438; ** ***p*** ** = 0.025**	−0.186; *p* = 0.364	−0.329; *p* = 0.101	−0.151; *p* = 0.462
Glucose	**0.499; ** ***p*** ** = 0.009**	−0.045; *p* = 0.828	−0.056; *p* = 0.785	0.200; *p* = 0.327	−0.304; *p* = 0.131	0.188; *p* = 0.357
ADMA	−0.187; *p* = 0.113	0.304; *p* = 0.139	**0.689; ** ***p*** **<0.001**	0.262; *p* = 0.206	**0.615; ** ***p*** **<0.001**	0.128; *p* = 0.540
sVCAM-1	−0.209; *p* = 0.088	−0.352; *p* = 0.118	**0.374; ** ***p*** ** = 0.002**	0.058; *p* = 0.8	**0.441; ** ***p*** **<0.001**	0.188; *p* = 0.414
hsCRP	**−0.471; ** ***p*** **<0.001**	−0.289; *p* = 0.229	**0.445; ** ***p*** **<0.001**	0.137; *p* = 0.576	**0.557; ** ***p*** **<0.001**	0.254; *p* = 0.293
IL-6	0.122; *p* = 0.609	0.289; *p* = 0.17	**0.468; ** ***p*** ** = 0.038**	**0.529; ** ***p*** ** = 0.008**	**0.471; ** ***p*** ** = 0.036**	**0.412; ** ***p*** ** = 0.045**
sTNFRII	−0.56; *p* = 0.816	−0.046; *p* = 0.831	**0.568; ** ***p*** ** = 0.009**	**0.576; ** ***p*** ** = 0.003**	**0.567; ** ***p*** ** = 0.009**	**0.559; ** ***p*** ** = 0.005**
D-dimer	−0.069; *p* = 0.772	0.048; *p* = 0.824	0.349; *p* = 0.132	0.188; *p* = 0.379	0.394; *p* = .086	0.139; *p* = 0.518

BMI = Body mass index; TChol: Total cholesterol.

## Discussion

In the present study, we investigated the association among sTWEAK and sCD163 plasma concentrations, the sCD163/sTWEAK ratio and other inflammatory, endothelial dysfunction and thrombotic biomarkers (hsCRP, IL-6, sTNFRII, sVCAM-1, ADMA and D-dimer) with the presence of HIV infection. We also investigated the impact of ART on these biomarkers. We observed that HIV-infected patients had decreased plasma levels of sTWEAK and increased concentrations of sCD163, hsCRP, IL-6, sTNFRII, sVCAM-1 and ADMA compared with healthy subjects. ART decreased the levels of sCD163, sVCAM-1, hsCRP, IL-6 and sTNFRII but did not affect sTWEAK and ADMA concentrations, reflecting a reduction in inflammation and immune activation associated with HIV.

Several pro-inflammatory mediators and adhesion molecules implicated in the progression of atherosclerosis, including hsCRP, IL-6, sVCAM-1, sICAM-1, P-selectin, E-selectin and ADMA, are elevated in HIV-infected patients [34]-[36]. IL-6, hsCRP, and D-dimer predict risk for CVD and all-cause mortality in HIV-infected patients [37]–[38]. The soluble TNF receptors levels have been also associated with the HIV-disease progression [39]. The sVCAM-1 levels are associated with carotid intima media thickness [40], and ADMA is associated with coronary artery calcium scores [41]. Both sVCAM-1 and ADMA are surrogate markers of atherosclerosis. In agreement with these observations, our patients showed increased hsCRP, IL-6, sTNFRII, sVCAM-1 and ADMA plasma concentrations. In addition to these traditional biomarkers, we evaluated the sCD163 plasma concentrations and, for the first time, characterized sTWEAK and the sCD163/sTWEAK ratio in patients with HIV.

Previous studies have demonstrated that sTWEAK and sCD163 levels are independently associated with cardiovascular disease. sTWEAK plasma concentrations are lower and sCD163 levels are higher in patients with carotid, peripheral and coronary artery disease [12]–[14], [28]. Moreover, circulating sCD163 and sTWEAK are both biomarkers of subclinical atherosclerosis in asymptomatic subjects, independent of traditional cardiovascular risk factors [24]. sTWEAK and the sCD163/sTWEAK ratio have also been associated with long-term global and cardiovascular mortality in patients with peripheral artery disease [22]. We observed that treatment-naive HIV patients had decreased sTWEAK plasma levels and increased concentrations of sCD163 compared with healthy subjects. This is the first study analyzing sTWEAK in HIV-infected patients. The mechanisms leading to lower sTWEAK levels in these subjects are not known. However, decreased sTWEAK plasma concentration has been also reported in other inflammatory pathologies, including rheumatoid arthritis, atherosclerosis and chronic kidney disease [16]–[18]. A number of studies have reported that the pathological effects of TWEAK are mediated by the binding of TWEAK with its receptor Fn14 [42]. Fn14 expression is increased under inflammatory conditions in multiple tissues [43]. Therefore, the decreased sTWEAK levels observed in our study could reflect an enhanced uptake by Fn14 in inflamed tissues. Although it is not entirely clear [44], it seems that sTWEAK binds to CD163 [23]. Therefore, sTWEAK levels may be also decreased by clearance via its scavenger receptor CD163 [24]. Increased CD163 expression has been reported in monocytes from HIV-infected subjects [45]. On this basis, we hypothesized that both mechanisms, an increased expression of Fn14 and an upregulation of CD163 on monocyte/macrophages, should be involved in the decreased sTWEAK levels observed in HIV-infected patients.

Increased sCD163 plasma levels in HIV-infected patients have been reported in previous studies. Hearps et al. demonstrated a higher concentration of sCD163 in 38 young viremic HIV-positive patients compared with HIV-uninfected controls [46]. In another study, sCD163 levels were elevated in 30 patients with chronic HIV infection and 14 patients with early HIV infection compared with 29 HIV-seronegative individuals [30]. In contrast to these studies and our results, a previous report comparing 11 uninfected donors and 38 HIV-infected patients found no differences in the sCD163 levels, although 23 patients were receiving ART [45]. In our study, the sCD163 levels and the sCD163/sTWEAK ratio were inversely associated with CD4 nadir at baseline and with CD4 count and the CD4/CD8 ratio after ART. Tippet et al. described an association between sCD163 and CD4 count [45], and Burdo et al. reported a similar association between sCD163 and the CD4/CD8 ratio [31]. However, although our results have been confirmed in these previous studies, we must be cautious with these associations due to our relatively small sample size.

sCD163 and the sCD163/sTWEAK ratio decreased significantly after ART, but no changes were observed for sTWEAK. Consistent with our results, Burdo et al. reported a decrease in sCD163 plasma levels after 3 months of ART in chronically infected and early diagnosed HIV patients [31]. In chronically infected HIV patients, sCD163 levels remained elevated compared with control subjects. Additionally, in early diagnosed HIV patients, the sCD163 concentration returned to levels observed in HIV-seronegative individuals [30]. Higher levels of sCD163 in HIV-infected patients with ART have also been reported in some but not all studies [31], [46].

We observed a reduction in hsCRP, IL-6, sTNFRII and sVCAM-1 after ART, but no reduction was found in ADMA or D-dimer levels. Previous studies have also described an improvement in these inflammatory and/or endothelial dysfunction biomarkers after ART. Thus, a decrease in sVCAM-1, sICAM-1, E-selectin and hsCRP was observed in 115 HIV-infected patients after 2 and 14 months of antiretroviral treatment [47]. In a further study, TNF-α, sTNFRI, sTNFRII sVCAM-1 and sICAM-1 plasma levels decreased significantly after 24 and 96 weeks of ART [48]. Moreover, D-dimer levels were also found decreased with initiation of ART [49], [50]. In contrast to our results, a recent report based on patients in the SMART study showed a decrease in ADMA levels after 12 months of ART; differences were only observed in participants randomized to immediate ART but not in patients randomized to deferral of ART [51]. Therefore, differences in ADMA levels due to ART require further research.

In our study, in spite of ART, sCD163 plasma levels remained elevated in HIV-infected patients compared with control subjects. Higher levels of sCD163 in HIV-infected patients with ART have also been reported in other studies [31], [32], [46], [52]. This result suggests that in spite of controlled viral replication via ART, HIV patients may have a chronic immune activation state that cannot be completely abrogated by ART. In this line, it has been recently proposed that HIV infection trigger immune changes which, if persist through time, may not be completely resolved despite controlling the initial stimulus (HIV infection) [53]. In this sense, there are evidences supporting that the timing of initiating ART may be important to control immune activation. Burdo et al. demonstrated that ART initiated within 1 year of infection (early diagnosed HIV patients) returned sCD163 levels to observed in HIV-seronegative individuals, but not if started later (chronically infected HIV patients) [30]. Moreover, our results show that hsCRP, sTNFRII and ADMA remained elevated in spite of ART, reflecting persistent inflammation and endothelial dysfunction, as previously reported in patients receiving ART [34], [47], [54]. However, we cannot exclude other potential underlying mechanisms responsible of sCD163 elevation in ART treated HIV patients, such as residual undetected HIV replication, increased translocation of bacterial products from the gastrointestinal tract due to mucosal injury, coinfections such as cytomegalovirus or HCV and the presence of established atherosclerotic lesions. Altogether, chronic immune activation, inflammation and endothelial dysfunction may explain the increased cardiovascular risk observed in these patients even when receiving proper treatment [55].

In our study, sCD163 plasma concentrations and the sCD163/sTWEAK ratio correlated with some inflammatory and endothelial biomarkers in treatment-naive HIV patients, but not after 48 weeks of ART. These results suggest a different effect of ART on monocyte activation and inflammation or endothelial dysfunction. The decrease in sCD163 with ART was modulated by the type of drug used. Thus, patients who were taking PIs had a lower decrease in sCD163 concentration compared with those without PIs. This finding has not been reported previously and could suggest that PIs, despite their antiviral efficacy, may have less effect on controlling monocyte-macrophage activation. Consistent with this suggestion, the relationship between PIs and cardiovascular disease in HIV infected patients is well recognized [6], [56], [57].

HIV-infected patients receiving ART who had HCV-HIV co-infection and/or persistent HIV replication presented a higher plasma concentration of sCD163 and sCD163/sTWEAK ratio. To our knowledge, there are no previous data related to HCV-HIV co-infection and sCD163. Our findings may indicate that the failure of ART to control viral replication or the existence of HCV-HIV co-infection attenuates the beneficial effect of this therapy on inflammation and macrophage activation. Thus, there may be an increased cardiovascular risk in these patients. In support of this hypothesis, previous data have indicated the importance of viral replication control in vascular disease [5]. Moreover, in a cross-sectional study, Sosner et al. reported that the prevalence of subclinical atherosclerosis was significantly higher in HCV-HIV co-infected patients. The increased atherosclerosis was independent of traditional risk factors, such as LDL-cholesterol and blood pressure [58]. Therefore, the presence of either HCV-HIV co-infection and/or active viral replication may justify a more careful cardiovascular risk management.

One limitation of this study is the relatively low number of patients. However, the strict inclusion criteria for patients and the design of our study limited us to include more VIH patients. Moreover, it is important to note that in the present work we could not analyze the effect mediated by HCV coinfection and/or persistence of HIV replication on immune activation in an independent manner. However, these data raise an attractive hypothesis to be explored in future studies.

In conclusion, we demonstrate that sTWEAK levels are reduced, whereas sCD163 and the sCD163/sTWEAK ratio are increased in HIV patients compared with healthy subjects. ART diminishes sCD163 levels in HIV patients, but no effect is observed for sTWEAK. Our study suggests that treatment with PIs or the presence of HCV-HIV co-infection and/or active HIV replication attenuates the beneficial effects of ART on immune activation, thereby facilitating atherosclerosis.

## References

[1] Mocroft A, Reiss P, Gasiorowski J, Ledergerber B, Kowalska J, et al (2010) Serious fatal and nonfatal non-AIDS-defining illnesses in Europe. J Acquir Immune Defic Syndr 55: 262–70. 20700060 10.1097/QAI.0b013e3181e9be6b

[2] Currier JS, Lundgren JD, Carr A, Klein D, Sabin CA, et al (2008) Epidemiological evidence for cardiovascular disease in HIV-infected patients and relationship to highly active antiretroviral therapy. Circulation 118: e29–35. 18566319 10.1161/CIRCULATIONAHA.107.189624 PMC5153327

[3] Triant VA, Lee H, Hadigan C, Grinspoon SK (2007) Increased acute myocardial infarction rates and cardiovascular risk factors among patients with human immunodeficiency virus disease. J Clin Endocrinol Metab 92: 2506–12. 17456578 10.1210/jc.2006-2190 PMC2763385

[4] Baker JV, Lundgren JD (2011) Cardiovascular implications from untreated human immunodeficiency virus infection. Eur Heart J 32: 945–51. 21228007 10.1093/eurheartj/ehq483 PMC3076665

[5] El-Sadr WM, Lundgren J, Neaton JD, Gordin F, Abrams D, et al (2006) CD4+ count-guided interruption of antiretroviral treatment. N Engl J Med 355: 2283–96. 17135583 10.1056/NEJMoa062360

[6] Dubé MP, Lipshultz SE, Fichtenbaum CJ, Greenberg R, Schecter AD, et al (2008) Effects of HIV infection and antiretroviral therapy on the heart and vasculature. Circulation 118: e36–40. 18566318 10.1161/CIRCULATIONAHA.107.189625

[7] Law MG, Friis-Møller N, El-Sadr WM, Weber R, Reiss P, et al (2006) The use of the Framingham equation to predict myocardial infarctions in HIV-infected patients: comparison with observed events in the D:A:D Study. HIV Med 7: 218–30. 16630034 10.1111/j.1468-1293.2006.00362.x

[8] Chicheportiche Y, Bourdon PR, Xu H, Hsu YM, Scott H, et al (1997) TWEAK, a new secreted ligand in the tumor necrosis factor family that weakly induces apoptosis. J Biol Chem 272: 32401–10. 9405449 10.1074/jbc.272.51.32401

[9] Blanco-Colio LM, Martín-Ventura JL, Munoz-Garcia B, Moreno JA, Meilhac O, et al (2007) TWEAK and Fn14. New players in the pathogenesis of atherosclerosis. Front Biosci 12: 3648–55. 17485328 10.2741/2341

[10] Nakayama M, Harada N, Okumura K, Yagita H (2003) Characterization of murine TWEAK and its receptor (Fn14) by monoclonal antibodies. Biochem Biophys Res Commun 306: 819–25. 12821115 10.1016/s0006-291x(03)01051-9

[11] Kim SH, Kang YJ, Kim WJ, Woo DK, Lee Y, et al (2004) TWEAK can induce pro-inflammatory cytokines and matrix metalloproteinase-9 in macrophages. Circ J 68: 396–9. 15056843 10.1253/circj.68.396

[12] Moreno JA, Dejouvencel T, Labreuche J, Smadja DM, Dussiot M, et al (2010) Peripheral artery disease is associated with a high CD163/TWEAK plasma ratio. Arterioscler Thromb Vasc Biol 30: 1253–62. 20299688 10.1161/ATVBAHA.110.203364

[13] Jelić-Ivanović Z, Bujisić N, Spasić S, Bogavac-Stanojević N, Spasojević-Kalimanovska V, et al (2009) Circulating sTWEAK improves the prediction of coronary artery disease. Clin Biochem 42: 1381–6. 19505454 10.1016/j.clinbiochem.2009.06.001

[14] Blanco-Colio LM, Martín-Ventura JL, Muñóz-García B, Orbe J, Páramo JA, et al (2007) Identification of soluble tumor necrosis factor-like weak inducer of apoptosis (sTWEAK) as a possible biomarker of subclinical atherosclerosis. Arterioscler Thromb Vasc Biol 27: 916–22. 17272745 10.1161/01.ATV.0000258972.10109.ff

[15] Martín-Ventura JL, Lindholt JS, Moreno JA, Vega de Céniga M, Meilhac O, et al (2011) Soluble TWEAK plasma levels predict expansion of human abdominal aortic aneurysms. Atherosclerosis 214: 486–9. 21130992 10.1016/j.atherosclerosis.2010.11.009

[16] Kralisch S, Ziegelmeier M, Bachmann A, Seeger J, Lössner U, et al (2008) Serum levels of the atherosclerosis biomarker sTWEAK are decreased in type 2 diabetes and end-stage renal disease. Atherosclerosis 199: 440–4. 18054361 10.1016/j.atherosclerosis.2007.10.022

[17] Carrero JJ, Ortiz A, Qureshi AR, Martín-Ventura JL, Bárány P, et al (2009) Additive effects of soluble TWEAK and inflammation on mortality in hemodialysis patients. Clin J Am Soc Nephrol 4: 110–8. 18945991 10.2215/CJN.02790608 PMC2615702

[18] Valdivielso JM, Coll B, Martín-Ventura JL, Moreno JA, Egido J, et al (2013) Soluble TWEAK is associated with atherosclerotic burden in patients with chronic kidney disease. J Nephrol 26: 1105–13. 23475462 10.5301/jn.5000245

[19] Chorianopoulos E, Rosenberg M, Zugck C, Wolf J, Katus HA, et al (2009) Decreased soluble TWEAK levels predict an adverse prognosis in patients with chronic stable heart failure. Eur J Heart Fail 11: 1050–6. 19875405 10.1093/eurjhf/hfp139

[20] Chorianopoulos E, Jarr K, Steen H, Giannitsis E, Frey N, et al (2010) Soluble TWEAK is markedly upregulated in patients with ST-elevation myocardial infarction and related to an adverse short-term outcome. Atherosclerosis 211: 322–6. 20303491 10.1016/j.atherosclerosis.2010.02.016

[21] Yilmaz MI, Sonmez A, Ortiz A, Saglam M, Kilic S, et al (2011) Soluble TWEAK and PTX3 in nondialysis CKD patients: impact on endothelial dysfunction and cardiovascular outcomes. Clin J Am Soc Nephrol 6: 785–92. 21330486 10.2215/CJN.09231010 PMC3069370

[22] Urbonaviciene G, Martin-Ventura JL, Lindholt JS, Urbonavicius S, Moreno JA, et al (2011) Impact of soluble TWEAK and CD163/TWEAK ratio on long-term cardiovascular mortality in patients with peripheral arterial disease. Atherosclerosis 219: 892–9. 21962403 10.1016/j.atherosclerosis.2011.09.016

[23] Bover LC, Cardó-Vila M, Kuniyasu A, Sun J, Rangel R, et al (2007) A previously unrecognized protein-protein interaction between TWEAK and CD163: potential biological implications. J Immunol 178: 8183–94. 17548657 10.4049/jimmunol.178.12.8183

[24] Moreno JA, Muñoz-García B, Martín-Ventura JL, Madrigal-Matute J, Orbe J, et al (2009) The CD163-expressing macrophages recognize and internalize TWEAK: potential consequences in atherosclerosis. Atherosclerosis 207: 103–10. 19473660 10.1016/j.atherosclerosis.2009.04.033

[25] Møller HJ, Peterslund NA, Graversen JH, Moestrup SK (2002) Identification of the hemoglobin scavenger receptor/CD163 as a natural soluble protein in plasma. Blood 99: 378–80. 11756196 10.1182/blood.v99.1.378

[26] Davis BH, Zarev PV (2005) Human monocyte CD163 expression inversely correlates with soluble CD163 plasma levels. Cytometry B Clin Cytom 63: 16–22. 15624200 10.1002/cyto.b.20031

[27] Moreno JA, Ortega-Gómez A, Delbosc S, Beaufort N, Sorbets E, et al (2012) In vitro and in vivo evidence for the role of elastase shedding of CD163 in human atherothrombosis. Eur Heart J 33: 252–63. 21606088 10.1093/eurheartj/ehr123

[28] Aristoteli LP, Møller HJ, Bailey B, Moestrup SK, Kritharides L (2006) The monocytic lineage specific soluble CD163 is a plasma marker of coronary atherosclerosis. Atherosclerosis 184: 342–7. 15979079 10.1016/j.atherosclerosis.2005.05.004

[29] Levy AP, Purushothaman KR, Levy NS, Purushothaman M, Strauss M, et al (2007) Downregulation of the hemoglobin scavenger receptor in individuals with diabetes and the Hp 2-2 genotype: implications for the response to intraplaque haemorrhage and plaque vulnerability. Circ Res 101: 106–10. 17525367 10.1161/CIRCRESAHA.107.149435

[30] Burdo TH, Lentz MR, Autissier P, Krishnan A, Halpern E, et al (2011) Soluble CD163 made by monocyte/macrophages is a novel marker of HIV activity in early and chronic infection prior to and after anti-retroviral therapy. J Infect Dis 204: 154–63. 21628670 10.1093/infdis/jir214 PMC3105035

[31] Burdo TH, Lo J, Abbara S, Wei J, DeLelys ME, et al (2011) Soluble CD163, a novel marker of activated macrophages, is elevated and associated with noncalcified coronary plaque in HIV-infected patients. J Infect Dis 204: 1227–36. 21917896 10.1093/infdis/jir520 PMC3203384

[32] Subramanian S, Tawakol A, Burdo TH, Abbara S, Wei J, et al (2012) Arterial inflammation in patients with HIV. JAMA 308: 379–86. 22820791 10.1001/jama.2012.6698 PMC3724172

[33] Panel de expertos de Gesida y Plan Nacional sobre el Sida (2012) [Consensus document of Gesida and Spanish Secretariat for the National Plan on AIDS (SPNS) regarding combined antiretroviral treatment in adults infected by the human immunodeficiency virus (January 2012)]. Enferm Infecc Microbiol Clin 30: e1–89. 22633764 10.1016/j.eimc.2012.03.006

[34] Neuhaus J, Jacobs DR Jr, Baker JV, Calmy A, Duprez D, et al (2010) Markers of inflammation, coagulation, and renal function are elevated in adults with HIV infection. J Infect Dis 201: 1788–95. 20446848 10.1086/652749 PMC2872049

[35] Kurz K, Teerlink T, Sarcletti M, Weiss G, Zangerle R, et al (2009) Plasma concentrations of the cardiovascular risk factor asymmetric dimethylarginine (ADMA) are increased in patients with HIV-1 infection and correlate with immune activation markers. Pharmacol Res 60: 508–14. 19651212 10.1016/j.phrs.2009.07.009

[36] Calza L, Pocaterra D, Pavoni M, Colangeli V, Manfredi R, et al (2009) Plasma levels of VCAM-1, ICAM-1, E-Selectin, and P-Selectin in 99 HIV-positive patients versus 51 HIV-negative healthy controls. J Acquir Immune Defic Syndr 50: 430–2. 19322038 10.1097/QAI.0b013e31819a292c

[37] Kuller LH, Tracy R, Belloso W, De Wit S, Drummond F, et al (2008) Inflammatory and coagulation biomarkers and mortality in patients with HIV infection. PLoS Med 5: e203. 18942885 10.1371/journal.pmed.0050203 PMC2570418

[38] Triant VA, Meigs JB, Grinspoon SK (2009) Association of C-reactive protein and HIV infection with acute myocardial infarction. J Acquir Immune Defic Syndr 51: 268–73. 19387353 10.1097/QAI.0b013e3181a9992c PMC2763381

[39] Kalayjian RC, Machekano RN, Rizk N, Robbins GK, Gandhi RT, et al (2010) Pretreatment levels of soluble cellular receptors and interleukin-6 are associated with HIV disease progression in subjects treated with highly active antiretroviral therapy. J Infect Dis 201: 1796–805. 20446847 10.1086/652750 PMC2873127

[40] Ross AC, Rizk N, O'Riordan MA, Dogra V, El-Bejjani D, et al (2009) Relationship between inflammatory markers, endothelial activation markers, and carotid intima-media thickness in HIV-infected patients receiving antiretroviral therapy. Clin Infect Dis 49: 1119–27. 19712036 10.1086/605578 PMC3895473

[41] Jang JJ, Berkheimer SB, Merchant M, Krishnaswami A (2011) Asymmetric dimethylarginine and coronary artery calcium scores are increased in patients infected with human immunodeficiency virus. Atherosclerosis 217: 514–7. 21549379 10.1016/j.atherosclerosis.2011.03.035

[42] Muñoz-García B, Moreno JA, López-Franco O, Sanz AB, Martín-Ventura JL, et al (2009) Tumor necrosis factor-like weak inducer of apoptosis (TWEAK) enhances vascular and renal damage induced by hyperlipidemic diet in ApoE-knockout mice. Arterioscler Thromb Vasc Biol 29: 2061–8. 19778942 10.1161/ATVBAHA.109.194852

[43] Muñoz-García B, Martín-Ventura JL, Martínez E, Sánchez S, Hernández G, et al (2006) Fn14 is upregulated in cytokine-stimulated vascular smooth muscle cells and is expressed in human carotid atherosclerotic plaques: modulation by atorvastatin. Stroke 37: 2044–53. 16809572 10.1161/01.STR.0000230648.00027.00

[44] Fick A, Lang I, Schäfer V, Seher A, Trebing J, et al (2012) Studies of binding of tumor necrosis factor (TNF)-like weak inducer of apoptosis (TWEAK) to fibroblast growth factor inducible 14 (Fn14). J Biol Chem. 287: 484–95. 22081603 10.1074/jbc.M111.287656 PMC3249102

[45] Tippett E, Cheng WJ, Westhorpe C, Cameron PU, Brew BJ, et al (2011) Differential expression of CD163 on monocyte subsets in healthy and HIV-1 infected individuals. PLoS One 6: e19968. 21625498 10.1371/journal.pone.0019968 PMC3098854

[46] Hearps AC, Maisa A, Cheng WJ, Angelovich TA, Lichtfuss GF, et al (2012) HIV infection induces age-related changes to monocytes and innate immune activation in young men that persist despite combination antiretroviral therapy. AIDS 26: 843–53. 22313961 10.1097/QAD.0b013e328351f756

[47] Kristoffersen US, Kofoed K, Kronborg G, Giger AK, Kjaer A, et al (2009) Reduction in circulating markers of endothelial dysfunction in HIV-infected patients during antiretroviral therapy. HIV Med 10: 79–87. 19200170 10.1111/j.1468-1293.2008.00661.x

[48] McComsey GA, Kitch D, Daar ES, Tierney C, Jahed NC, et al (2012) Inflammation markers after randomization to abacavir/lamivudine or tenofovir/emtricitabine with efavirenz or atazanavir/ritonavir. AIDS 26: 1371–85. 22546988 10.1097/QAD.0b013e328354f4fb PMC3560932

[49] Funderburg NT, Andrade A, Chan ES, Rosenkranz SL, Lu D, et al (2013) Dynamics of Immune Reconstitution and Activation Markers in HIV+ Treatment-Naïve Patients Treated with Raltegravir, Tenofovir Disoproxil Fumarate and Emtricitabine. PLoS One 8: e83514. 24367599 10.1371/journal.pone.0083514 PMC3867440

[50] Jong E, Louw S, van Gorp EC, Meijers JC, ten Cate H, et al (2010) The effect of initiating combined antiretroviral therapy on endothelial cell activation and coagulation markers in South African HIV-infected individuals. Thromb Haemost 104: 1228–34. 20886182 10.1160/TH10-04-0233

[51] Baker JV, Neuhaus J, Duprez D, Freiberg M, Bernardino JI, et al (2012) HIV replication, inflammation, and the effect of starting antiretroviral therapy on plasma asymmetric dimethylarginine, a novel marker of endothelial dysfunction. J Acquir Immune Defic Syndr 60: 128–34. 22421746 10.1097/QAI.0b013e318252f99f PMC3360839

[52] Martin GE, Gouillou M, Hearps AC, Angelovich TA, Cheng AC, et al (2013) Age-associated changes in monocyte and innate immune activation markers occur more rapidly in HIV infected women. PLoS One 8: e55279. 23365694 10.1371/journal.pone.0055279 PMC3554695

[53] Sandler NG, Sereti I (2014) Can early therapy reduce inflammation? Curr Opin HIVAIDS 9: 72–9. 10.1097/COH.0000000000000020 PMC4160125 24247669

[54] Hsue PY, Scherzer R, Hunt PW, Schnell A, Bolger AF, et al.. (2012) Carotid Intima-Media Thickness Progression in HIV-Infected Adults Occurs Preferentially at the Carotid Bifurcation and Is Predicted by Inflammation. J Am Heart Assoc pii: jah3-e000422. 10.1161/JAHA.111.000422 PMC3487373 23130122

[55] Islam FM, Wu J, Jansson J, Wilson DP (2012) Relative risk of cardiovascular disease among people living with HIV: a systematic review and meta-analysis. HIV Med 13: 453–468. 22413967 10.1111/j.1468-1293.2012.00996.x

[56] Stein JH, Klein MA, Bellehumeur JL, McBride PE, Wiebe DA, et al (2001) Use of human immunodeficiency virus-1 protease inhibitors is associated with atherogenic lipoprotein changes and endothelial dysfunction. Circulation 104: 257–62. 11457741 10.1161/01.cir.104.3.257

[57] Worm SW, Sabin C, Weber R, Reiss P, El-Sadr W, et al (2010) Risk of myocardial infarction in patients with HIV infection exposed to specific individual antiretroviral drugs from the 3 major drug classes: the data collection on adverse events of anti-HIV drugs (D:A:D) study. J Infect Dis 201: 318–30. 20039804 10.1086/649897

[58] Sosner P, Wangermez M, Chagneau-Derrode C, Le Moal G, Silvain C (2012) Atherosclerosis risk in HIV-infected patients: the influence of hepatitis C virus co-infection. Atherosclerosis 222: 274–7. 22417840 10.1016/j.atherosclerosis.2012.02.027

